# Hybrids without hybridization—can it revolutionize horticulture?

**DOI:** 10.1038/s41438-018-0113-3

**Published:** 2018-12-21

**Authors:** Steve van Nocker

**Affiliations:** Michigan State University, Michigan, USA

The often-cited idiom “The apple doesn’t fall far from the tree” refers to the occasional striking similarity in appearance or behavior between a human parent and child. However, in heterozygous organisms—be it human or apple—strong similarity in multiple traits across generations tends to be the exception. Rather, the phenomenon of genetic segregation ensures that offspring show variation. While this can be advantageous in nature, it poses numerous problems for horticulture and plant agriculture in general, as the perpetual need to reproduce high-performing hybrids through crossing of parental genotypes, as well as the clonal vegetative propagation of elite hybrid cultivars, involves expensive and time consuming procedures. A recent study by Khanday and colleagues, published 12 December in the journal *Nature*^1^, combined the characterization of a mechanism that triggers embryogenesis in the absence of fertilization, with a previously identified strategy to bypass meiosis, to demonstrate the feasibility of inducing apomixis—asexual reproduction through seeds—of hybrid plants.

This work had roots in a previous molecular study of somatic embryogenesis—the phenomenon by which an embryo is generated from differentiated, somatic cells—in *Brassica napus*. In an analysis of genes expressed very early during somatic embryogenesis, Boutilier and colleagues had identified the *BABY BOOM* (*BBM*) gene, which encodes one of the largest assortments of AP2/ERF class of transcription factors found in plants^2^. Its early expression and identity as a transcription factor suggested that it might have a regulatory role, and indeed, ectopic expression of *BBM* resulted in spontaneous embryo formation in vegetative tissues in both Brassica and Arabidopsis^2^. Subsequently, Conner et al.^3^ characterized the function of *BBM* genes in *Pennisetum squamulatum*, where apomixis cosegregates with a locus containing multiple *BBM* copies. They found that transgenic expression of one of these genes in transgenic plants was sufficient to generate embryos in the absence of fertilization.

Khanday and colleagues adapted these findings to the study of the initiation of zygotic embryogenesis in rice. Rice contains several *BBM*-like genes^4^, and the authors first found that at least a subset of these genes were expressed in the rice zygote soon after fertilization. Also similar to findings in Brassica, one of these genes, *BBM1*, promoted formation of somatic embryos when expressed ectopically. These results were consistent with a role for *BBM1* in regulating early embryonic development, but a clue that *BBM1* might act as a trigger for embryogenesis came from earlier transcriptional profiling of zygotes, which suggested that *BBM1* was expressed only from the paternal genome^5^, which is contributed by the sperm. In the present study, the authors supported this by analyzing a genetic polymorphism distinguishing the maternal from paternal *BBM1* transcripts, and with the use of a *BBM1-GFP* marker gene, which was active in the zygote only when contributed from the male parent. Furthermore, the group showed that *BBM1* promotes its own expression in the zygote, thus potentially reinforcing its zygotic function.

The maternal copy of *BBM1* is also expressed in the embryo, but only after initiation of embryogenesis^1^. To explore a potential function for *BBM1* in initiating embryogenesis in the absence of other contributing paternal factors, the researchers expressed *BBM1* from the maternal genome in the egg cell, using the promoter regulatory sequence of a gene previously found to drive expression only in the egg cell in rice^6^. They observed embryo-like structures within ovules of the transgenic plants in the absence of fertilization, thus supporting an initiating role for *BBM1*. These structures failed to develop further, as was anticipated due to the absence of endosperm.

The investigators next asked if *BBM1* was necessary, as well as sufficient, for initiating embryogenesis. *BBM1* is a member of a small clade of AP2/ERF genes in rice^4^, with at least two others—*BBM2* and *BBM3*—also expressed in zygotes^1^. Since phylogenetically related genes may have overlapping and redundant function, the investigators used CRISPR-Cas9 technology and crossing to create double and triple mutants for these genes. A mutant lacking both *bbm1* and *bbm3* was fully fertile, showing the neither of these genes was required. However, *BBM1* function was found to be essential for full fertility in mutants lacking both *bbm2* and *bbm3*. This demonstrated that these three genes function redundantly.

In the absence of fertilization, embryos generated by maternal egg-cell specific expression of *BBM1* are expected to be haploid. Since artificially doubled haploid plants are often desirable in plant breeding and genomics^7^, the researchers then asked if these embryos could be rescued in the presence of endosperm. They found that by selfing the maternal-*BBM1* expressing line, viable seeds containing haploid embryos could be produced at relatively high frequency, thus demonstrating a novel approach for production of haploid plants.

Taken together, this research underscores the importance of the *BBM* family of genes in initiating zygotic embryogenesis in rice. A fundamental unanswered question is the mechanism by which *BBM1* initiates the programs of cell division and patterning associated with this process. The observed autoactivation of *BBM1* gene, as well as its subsequent activation of the maternal-*BBM1* copy, explains how a small amount of RNA or protein present in sperm might quickly establish a robust transcriptional program in the zygote. Presumably, this involves regulation of downstream genes that participate in patterning of the early embryo. In this way, BBM1 is functionally similar to mammalian pluripotency factors, such as Oct4, Sox2, and Nanog that regulate downstream key lineage-determining genes^8^. Defining the molecular pathways downstream of *BBM1* might therefore be relevant to attempts to reengineer plant tissues types and architectures for more efficient food, fiber, or biofuel production.

Khanday et al. then built further on their findings by investigating whether parthenogenic embryo formation could be linked with a recently identified strategy blocking meiosis, to produce embryos from diploid cells. Previous research in both rice and Arabidopsis showed that by disrupting three genes with key function in meiosis, diploid unrecombined gametes could be produced^9,10^. By combining mutations in these genes with transgenic maternal expression of *BBM1*, the investigators were then able to develop diploid, fertile, and apomictic lines, referred to as S-Apo (Synthetic-Apomictic).

This innovated work is a major step forward for breeding and production of hybrids. Practically, the observed similarity in function among *BBM* genes from dicot and monocot families suggests that the S-Apo system might be exploited for a variety of horticultural crop plants. Production of hybrid seed is a mainstay of the horticulture industry, especially for annual flowers and vegetables, in order to maintain traits and hybrid vigor, and the ability to propagate hybrids through seeds would have wide utility. However, challenges remain to further apply S-Apo in horticulture. The researchers point out that, although embryos are formed parthenogenically, fertilization is nevertheless required for endosperm development. Several genes that participate in limiting the development of endosperm in the absence of fertilization have been identified^11,12^, and manipulation of these genes, in combination with S-Apo, may produce viable seed in the absence of fertilization. This could have important implications for those horticultural crops, including most fruits and nuts, that may be negatively impacted by future decline in insect pollinators due to pesticide use and disease^13,14^.

These findings also have impact for horticultural crops that are currently propagated asexually through vegetative tissues. Maintaining clones of cultivars for long period of time typically requires extensive serial propagation, which can lead to accumulation of somatic mutations, viruses, and other pathogens. The ability to reproduce a genotype asexually via zygotic embryogenesis thus is a way around this. In addition, the potential to preserve the genotype through seeds—which are far more resilient—allows for very long term storage in seed banks.
